# How to Write Your First Clinical Case Report

**DOI:** 10.1016/j.jaccas.2022.09.016

**Published:** 2022-11-02

**Authors:** Julia Grapsa

**Figure:**
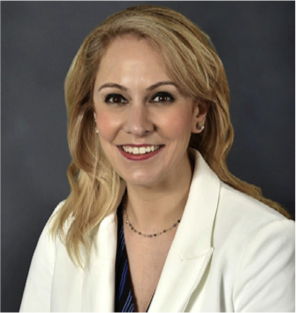

Do you remember when your first publication of a clinical case was? Most likely when you were a medical student or resident. Although the process for young authors is rewarding once the manuscript gets accepted, the writing and revision process can be exhausting. For some early career authors, it can be difficult to find a starting point. What is the main advice that the *JACC: Case Reports* editorial board can give you for publishing a clinical case? After having handled more than 9,000 submissions to the journal in the last 3 years, here is what we think.

## Select the Journal of Submission and Look at the Literature

Before you submit your manuscript, carefully peruse the potential journals of submission and read the author instructions for those journals. It is important to ensure that the journal of submission is the right one for your work. Carefully considering journal selection will allow you to avoid disappointment if the journal’s audience is not the best fit for your clinical case. Journals will also vary in styles and format offerings, so the first step should always be to visit the instructions for that particular journal to ensure that one of the formats is appropriate for your case.

Furthermore, before you begin to write the case, perform a comprehensive literature search. Just because it is new to you does not mean that the findings or topics are novel in the context of the published literature. A manuscript also reflects your work as a clinician, so make sure that you submit a case that best reflects your clinical achievements.

## Structure Your Report

Once you have selected the journal of submission, carefully reread the author instructions to structure your submission. The *JACC: Case Reports* authors instructions suggest a specific structure for a clinical case: history of presentation, physical examination, past medical history, differential diagnosis, investigations, management (medical/interventions), discussion, follow-up, conclusions, and learning objectives. For a clinical case, it is important to document the patient’s presentation and the physical examination. For example, in a patient with acute pulmonary embolism, it is important to document blood pressure, heart rate, and respiratory rate.

Another important component of the clinical case is the differential diagnosis, in which you should explain why you ended up with the final diagnosis. We do not wish to see a bullet point listing of possible diagnoses, but rather a narrative explanation of why you excluded other clinical issues. When you describe management, do not hesitate to describe any procedural complications. We learn from our mistakes, and a complication may be of great educational value for the medical community. When the *JACC: Case Reports* editorial board makes a call for procedural complications cases, we typically receive a decent number of malpractice cases. Therefore, make sure that you describe appropriate, guideline-directed clinical practice. Proper management can also a product of good mentorship, which I discuss later in this paper.

The Discussion section is crucial because it explains why your case merits publication. Why is it important to the medical community? Is it going to lead to further research that may eventually change clinical practice? Even if you did not follow clinical guidelines, this is important for us, because it is through clinical cases that we identify gaps in evidence that should be addressed.

Remember that there are also other formats for the journal, and you may choose a different format that better suits your case report.

## Include High-Quality Imaging

The foundation of a clinical case is imaging, and this should reflect the high quality standards of the *JACC* family of journals. As an interactive journal with audiovisual multimedia, do not forget to upload complementary videos when relevant to the reader’s understanding of the case. Investigations such as echocardiograms and angiograms are crucial for the management of the patient and should be included. Remember to remove any patient identifying or institutional information from figures and videos, detach background noise from videos, and upload the original mp4 file from your institution’s system. Video recordings of echocardiograms and angiography from your cell phone may detract from the quality of the videos.

## Request Senior Supervision as You Write and Revise Your Submission

Many submissions to the journal are rejected without review because of a lack of interest for the journal’s readership or a lack of sufficient novelty. However, rejection may often occur because the clinical case is poorly written if the junior author had not received oversight from the senior colleagues who are acknowledged in the manuscript. This is a learning process for junior authors: send it to your peers/senior coauthors with a deadline for comments, include those who have worked on the patient, and make sure that every author has read the manuscript and consented to the final version of submission. This is teamwork.

For early career authors, we are proud to have started the *JACC: Case Reports* Reviewer Mentoring Program, which aims to teach colleagues about the art of peer review, the publication process, and writing good cases and original research manuscripts. If you are interested, stay tuned for communications from the American College of Cardiology education team to apply to the next iteration of the program in 2023.

## Be Respectful in the Communications Surrounding Your Manuscript

It is respectful (and helpful) to the journal’s editors to provide a cover letter explaining why you feel that your clinical case merits publication. The cover letter can also include information about how the case might be accepted for presentation at an upcoming scientific meeting, requests for expedited review, or details about conflicts of interest.

Be careful to correctly write the name of the Editor-in-Chief. Personally, I often see my name being written Graspa rather than Grapsa. It is the legacy of my father—Greek and the surname means “writer.” Although we never reject a manuscript because an Editor-in-Chief’s surname is wrong or misspelled, this can hold up the processing of your paper and leave an impression on the editors that the authors did not take time and care to prepare all manuscript materials.

Furthermore, even though social media is commonly used in the medical community to discuss publications, this is not the proper channel for authors to submit a presubmission query. Messaging the editors on social media is unfair to the other authors who choose to submit their work for peer review. Addressing queries from authors on social media would also be unfair to the authors, because our decisions are group-led through our weekly editorial board meetings, and those messages are unsecure because they are housed on social media platforms. All presubmission queries should be sent to the Editorial Office (jacccr@acc.org), and staff members will triage any questions or issues to the editors.

We always ask our editors, reviewers, editorialists, and staff to be respectful in communications surrounding your manuscript. We make every effort to provide constructive feedback, even if the manuscript is rejected. With that in mind, we request that authors are also respectful of our editors, reviewers, editorialists, and all those working in the Editorial Office.

## Be Persistent: Do Not Get Disappointed or Discouraged

Even if your clinical case is rejected, it may not necessarily have been because of quality. It is possible there were reasons it did not reach priority for publication in that particular journal. For example, many other cases on that topic may already have been accepted/published. If the case has been accepted with revision after peer review, try to address the editors’ and reviewers’ comments and resubmit as soon as possible. A clinical case can easily get outdated if the authors delay submitting a revised version, particularly because of novelty concerns. If your case is rejected, do not get disappointed or discouraged. You may submit your case to another journal with a better outcome. Remember that we learn from a rejection letter, particularly if there are constructive comments included in the letter, and every positive or negative answer is an opportunity for us to grow.

## Submit Your Case: The Sky Is the Limit

As a last message, to all of the young authors who are trying to get their clinical cases published, remember that the “sky is the limit”—be creative, find a good mentor, and write up an interesting case. Once your manuscript is accepted, it is just the beginning of your academic career.

At *JACC: Case Reports*, we are always willing to help you through the submission process. Please send any presubmission queries to the Editorial Office: jacccr@acc.org. Furthermore, you may wish to watch the video featuring *JACC: Case Reports* Deputy Editor, Dr Mary Walsh, and Associate Editor, Dr David, discussing tips and tricks for how to write a successful clinical case.

